# Long-term effects on subclinical cardiovascular disease of switching from boosted protease inhibitors to dolutegravir

**DOI:** 10.1093/jac/dkad247

**Published:** 2023-09-05

**Authors:** Ana González-Cordón, Lambert Assoumou, Graeme Moyle, Laura Waters, Margaret Johnson, Pere Domingo, Julie Fox, Hans-Jürgen Stellbrink, Giovanni Guaraldi, Mar Masiá, Mark Gompels, Stephane De Wit, Eric Florence, Stefan Esser, François Raffi, Georg Behrens, Anton Pozniak, Jose M. Gatell, Esteban Martínez, on behalf of the NEAT 022 Study Group, Linos Vandekerckhove, Els Caluwé, Stephane De Wit, Coca Necsoi, Eric Florence, Maartje Van Frankenhuijsen, François Raffi, Clotilde Allavena, Véronique Reliquet, David Boutoille, Morane Cavellec, Elisabeth André-Garnier, Audrey Rodallec, Thierry Le Tourneau, Jérôme Connault, Jean-Michel Molina, Samuel Ferret, Miresta Previlon, Yazdan Yazdanpanah, Roland Landman, Véronique Joly, Adriana Pinto, Christine Katlama, Fabienne Caby, Nadine Ktorza, Schneider Luminita, Christoph Stephan, Timo Wolf, Gundolf Schüttfort, Juergen Rockstroh, Jan-Christian Wasmuth, Carolynne Schwarze-Zander, Christoph Boesecke, Hans-Jurgen Stellbrink, Christian Hoffmann, Michael Sabranski, Stephan Esser, Robert Jablonka, Heidi Wiehler, Georg Behrens, Matthias Stoll, Gerrit Ahrenstorf, Giovanni Guaraldi, Giulia Nardini, Barbara Beghetto, Antonella D’Arminio Montforte, Teresa Bini, Viola Cogliandro, Massimo Di Pietro, Francesco Maria Fusco, Massimo Galli, Stefano Rusconi, Andrea Giacomelli, Meraviglia Paola, Esteban Martinez, Ana González-Cordón, José Maria Gatell, Berta Torres, Pere Domingo, Gracia Mateo, Mar Gutierrez, Joaquin Portilla, Esperanza Merino, Sergio Reus, Vicente Boix, Mar Masia, Félix Gutiérrez, Sergio Padilla, Bonaventura Clotet, Eugenia Negredo, Anna Bonjoch, José L. Casado, Sara Bañón-Escandell, Jose Saban, Africa Duque, Daniel Podzamczer, Maria Saumoy, Laura Acerete, Juan Gonzalez-Garcia, José Ignacio Bernardino, José Ramón Arribas, Victor Hontañón, Graeme Moyle, Nicole Pagani, Margherita Bracchi, Jaime Vera, Amanda Clarke, Tanya Adams, Celia Richardson, Alan Winston, Borja Mora-Peris, Scott Mullaney, Laura Waters, Nahum de Esteban, Ana Milinkovic, Sarah Pett, Julie Fox, Juan Manuel Tiraboschi, Margaret Johnson, Mike Youle, Chloe Orkin, Simon Rackstraw, James Hand, Mark Gompels, Louise Jennings, Jane Nicholls, Sarah Johnston

**Affiliations:** Belgium; France; Germany; Italy; Spain; UK; 1 Hospital Clínic-IDIBAPS, University of Barcelona, Barcelona, Spain; 2 CIBER de Enfermedades Infecciosas (CIBERINFEC), Instituto de Salud Carlos III, Madrid, Spain; 3 INSERM, Institut Pierre Louis d’Épidémiologie et de Santé Publique, Sorbonne Université, Paris, France; 4 Consultant Physician in HIV Medicine, Chelsea and Westminster Hospital NHS Foundation Trust, London, UK; 5 Mortimer Market Centre, Central and North West London NHS Foundation Trust, London, UK; 6 Senior Consultant Physician in Thoracic Medicine, Royal Free London NHS Foundation Trust, London, UK; 7 Senior Consultant at Infectious Diseases Unit, Hospital de Sant Pau, Barcelona, Spain; 8 HIV Research Lead, Guy’s and St Thomas’ NHS Foundation Trust, London, UK; 9 Professor of Medicine, Infektionsmedizinisches Centrum, Hamburg, Germany; 10 Professor of Medicine, University of Modena and Reggio Emilia, Modena, Italy; 11 Professor of Medicine, Hospital General Universitario de Elche, Elche, Spain; 12 Clinical Lead for Allergy, Immunology and HIV, North Bristol NHS Trust, Bristol, UK; 13 Professor of Medicine, Centre Hospitalier Universitaire Saint-Pierre, Brussels, Belgium; 14 Head of the HIV Clinic, Universitair Ziekenhuis Antwerpen, Antwerp, Belgium; 15 Academic Director, Universitätsklinikum, Essen, Germany; 16 Professor of Infectious Diseases, Centre Hospitalier Universitaire, Nantes, France; 17 Profesor of Immunology, Medizinische Hochschule, Hannover, Germany; 18 Global Medical Director, ViiV Healthcare, Brentford, UK

## Abstract

**Background:**

In the NEAT022 trial, switching from boosted PIs (PI/r) to dolutegravir in people with HIV (PWH) with high cardiovascular risk decreased plasma lipids, soluble CD14 and adiponectin, and showed consistent favourable, although non-significant, effects on carotid intima-media thickness (CIMT) progression at 48 weeks. We hereby communicate planned final 96 week results on biomarker changes and CIMT progression.

**Methods:**

PWH on a PI/r-based triple therapy regimen were randomly assigned (1:1) to switch the PI/r component to dolutegravir either immediately (DTG-I group) or after 48 weeks (DTG-D group) and were followed up to 96 weeks. We assessed changes in biomarkers associated with inflammation, endothelial dysfunction, monocyte immune activation, oxidation, insulin resistance, hypercoagulability, heart failure, myocardial injury and glomerular and tubular kidney injury, and right and left CIMT progression at 48 and 96 weeks.

**Results:**

Of 415 PWH randomized, 287 (69%) and 143 (34%) contributed to the biomarker and CIMT substudies respectively. There were significant 96 week changes in biomarkers associated with inflammation, immune activation, oxidation, insulin resistance and myocardial injury. Most changes were favourable, except for adiponectin reduction, which may suggest higher insulin resistance. We were unable to detect significant changes in the progression of CIMT between arms or within arms at 96 weeks.

**Discussion:**

After 96 weeks, switching from PI/r to dolutegravir in PWH with high cardiovascular risk led to significant changes in several biomarkers associated with cardiovascular disease. Although most changes were favourable, adiponectin reduction was not. There were non-significant changes in CIMT progression.

## Introduction

Integrase inhibitors are currently the cornerstone of ART.^1^ Integrase inhibitors have shown a better lipid profile than other anchor drugs in first-line triple regimens.^2^ However, integrase inhibitors have been associated with higher weight gain than other antiretroviral classes.^3^ Weight gain may trigger metabolic derangements, ultimately leading to cardiovascular disease in the general population, and certainly this can also happen in persons with HIV (PWH) because their risk for cardiovascular disease exceeds that of the general population.^4^ Integrase inhibitors have been recently associated with a higher risk of diabetes, hypertension and cardiovascular events.^5–9^

Because of the similar virological outcomes expected with currently available regimens, antiretroviral switching trials are less likely than superiority trials to show clinical benefits, and greater attention should be placed on other potential advantages and disadvantages. The NEAT022 randomized clinical trial showed that switching from a ritonavir-boosted PI-based regimen to a dolutegravir-based regimen in virologically suppressed PWH with high cardiovascular disease risk was not only virologically non-inferior but also significantly improved plasma lipid profiles at 48^10^ and 96^11^ weeks. Weight gain was modest and limited to the first 48 weeks after dolutegravir initiation and was not associated with negative clinical metabolic outcomes at 96 weeks.^12^

Plasma biomarkers have contributed to better knowledge of the negative effects of uncontrolled HIV infection on the pathogenesis of cardiovascular disease^13^ and their reversal upon initiation of ART.^14,15^ Furthermore, HIV infection contributes to the progression of carotid artery intima-media thickness (CIMT)^16^ and ART may differentially impact on CIMT progression depending on the types of drugs used.^17^ Therefore, assessment of cardiovascular biomarkers and progression of CIMT seems justified in a trial switching ART to decrease plasma lipids and cardiovascular risk in virologically suppressed PWH with high cardiovascular risk.

We have previously reported that switching from boosted PIs to dolutegravir significantly decreased soluble CD14 and adiponectin,^18^ and showed consistent favourable, although non-significant, trends on common CIMT progression^19^ at 48 weeks. Whether the changes in biomarkers and CIMT progression after the first 48 weeks of dolutegravir initiation seen in the immediate switch (DTG-I group) are also reproduced in the deferred switch (DTG-D group) and whether they are maintained after 96 weeks of dolutegravir exposure is unknown. We hereby report planned final 96 week results on biomarker changes and CIMT progression in the NEAT022 trial.

## Methods

NEAT022 is an open-label, randomized, non-inferiority trial conducted in 32 clinical sites in six European countries. Briefly, eligible PWH older than 50 years, or older than 18 years with a 10 year Framingham cardiovascular disease risk score higher than 10%. They were required to be treated with a stable triple antiretroviral regimen consisting of a boosted PI plus two nucleoside reverse transcriptase analogues and to have a plasma HIV RNA of prior evidence of primary viral resistance to backbone nucleos(t)ides or previous episodes of documented virological failure were excluded. Eligible participants were randomly assigned (1:1) to switch the boosted-PI component to dolutegravir 50 mg/day, maintaining the two nucleos(t)ides unchanged, or to continue with the same boosted PI-based triple therapy regimen for 48 weeks and subsequently all patients in the boosted-PI arm were switched to dolutegravir and followed up to 96 weeks (DTG-D group)^11^ (Figures S1 and S2) (available as Supplementary data at *JAC* Online). The subclinical cardiovascular disease substudy protocol was approved by the ethics committees of all participating centres. All participants gave their written informed consent prior to study procedures. The study is registered on ClinicalTrials.gov (NCT02098837) and EudraCT (2013-003704-39).

We assessed markers associated with inflammation [high-sensitive C-reactive protein (hsCRP), IL-6], endothelial dysfunction [intercellular adhesion molecule 1 (ICAM-1), vascular cell adhesion molecule 1 (VCAM-1), E-selectin and P-selectin], monocyte immune activation [soluble CD14 (sCD14), soluble CD163 (sCD163)], oxidation [malondialdehyde (MDA), oxidized low-density lipoprotein (ox-LDL)], insulin resistance (insulin, adiponectin), hypercoagulability (D-dimer), myocardial injury [high-sensitive cardiac troponin T (hs-cTnT)], heart failure [N-terminal pro b-type natriuretic peptide (NT-proBNP)], kidney glomerular injury (cystatin C) and kidney tubular injury (urine β-2-microglobulin/creatinine ratio). Methods have been described in detail elsewhere.^18^

CIMT measurements procedures followed the Mannheim consensus.^19^ Briefly, measurements were performed on both common carotid arteries using a commercially validated ultrasound scanner equipped with a linear 7–10 MHz probe following a standardized procedure. CIMT was automatically assessed on the far wall of the common carotid artery at 1 cm from the bulb to avoid inter-individual variability.

The median percent changes from baseline in biomarkers and median changes in right and left CIMT at Weeks 48 and 96 in dolutegravir-based and PI-based arms were compared within arms and between arms with the non-parametric Mann–Whitney test.

## Results

Two hundred and eighty-seven (69%) participants contributed to the biomarker substudy and 143 (34%) participants contributed to the CIMT substudy at 96 weeks. Population characteristics in the biomarker and CIMT substudies did not differ between arms and matched those of the main study: >50 years, 88%; Framingham score 10%, 72%; men, 80%; Caucasian, 89%; median time with undetectable viral load, 4.2 years; current smokers, 55%; diabetes, 5%; family history of cardiovascular disease, 36%; hypertension, 26%; and lipid-lowering therapy, 32%.

**Figure 1:**
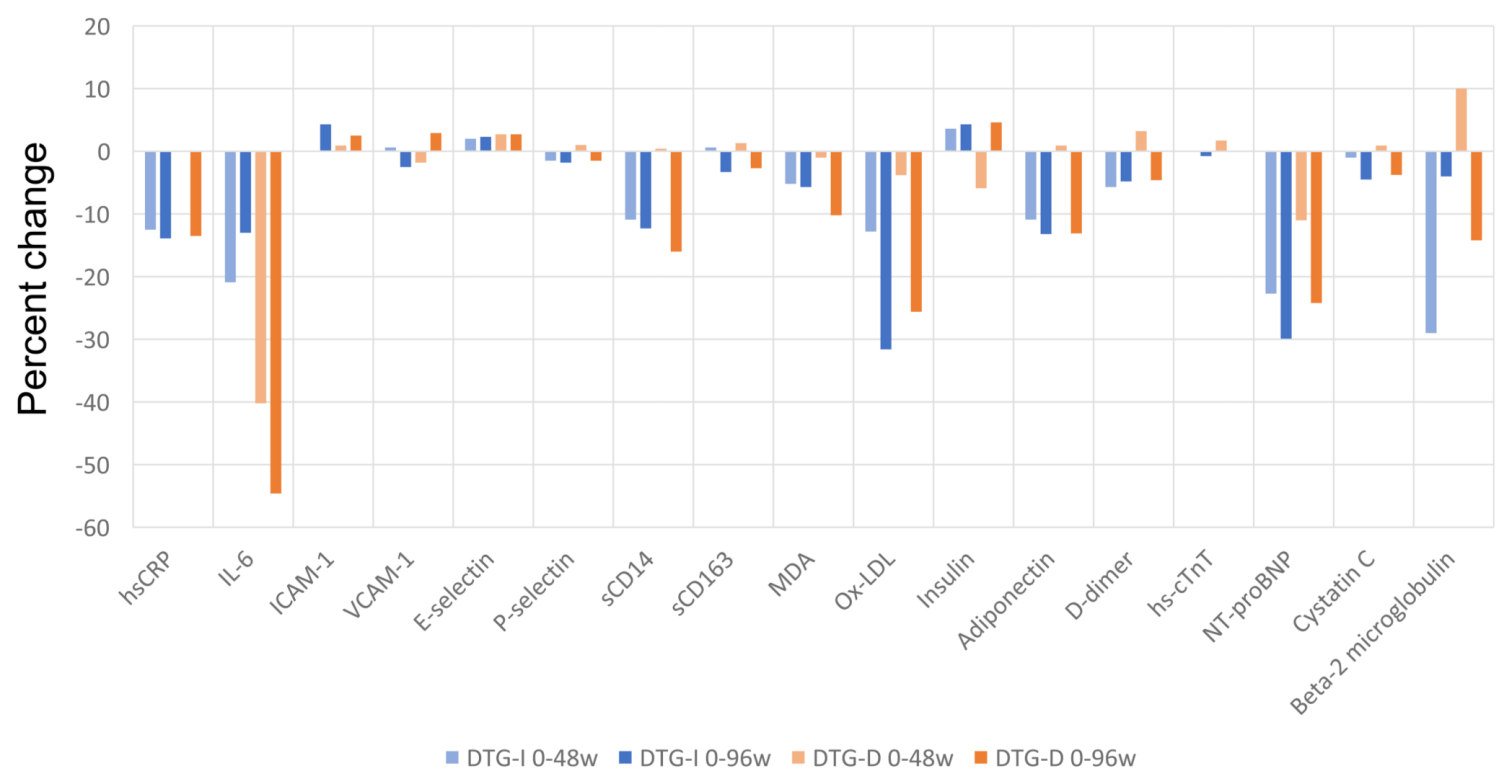
Median percent changes in selected biomarkers in DTG-I and DTG-D arms at 48 and 96 weeks. In the DTG-I arm, biomarker changes at 48 weeks were significant for hsCRP (−12.5%, *P* < 0.001), IL-6 (−20.9%, *P* = 0.024), sCD14 (−10.9%, *P* < 0.001), ox-LDL (−12.8%, *P* < 0.001), adiponectin (−10.9%, *P* < 0.001) and NT-proBNP (−22.7%, *P* < 0.001). In this arm, median percent changes at 96 weeks were significant for hsCRP (−13.9%, *P* < 0.001), IL-6 (−13%, *P* = 0.048), sCD14 (−12.3%, *P* < 0.001), ox-LDL (−31.6%, *P* < 0.001), adiponectin (−13.2%, *P* < 0.001) and NT-proBNP (−29.9%, *P* < 0.001). In the DTG-D arm, median percent changes in biomarkers at 48 weeks were significant for IL-6 (−40.2%, *P* = 0.048) only. In this arm, median percent changes at 96 weeks were significant for hsCRP (−13.5%, *P* < 0.001), IL-6 (−54.6%, *P* < 0.001), sCD14 (−16%, *P* < 0.001), ox-LDL (−25.6%, *P* < 0.001), adiponectin (−13.1%, *P* < 0.001) and NT-proBNP (−24.2%, *P* = 0.001). This figure appears in colour in the online version of *JAC* and in black and white in the print version of *JAC*.

We saw predominantly decreases in changes of biomarkers (Figure 1) with the strategy of switching from ritonavir-boosted PI-based to dolutegravir-based triple regimens in the NEAT022 study. At 48 weeks, differences between arms in percent changes of biomarkers were significant only for sCD14 (*P* < 0.001) and adiponectin (*P* < 0.001); at 96 weeks, there were no significant differences in percent changes of biomarkers between arms.

In the DTG-I arm, median percent biomarker changes at 48 weeks were significant for hsCRP (−12.5%, *P* < 0.001), IL-6 (−20.9%, *P* = 0.024), sCD14 (−10.9%, *P* < 0.001), ox-LDL (−12.8%, *P* < 0.001), adiponectin (−10.9%, <0.001) and NT-proBNP (−22.7%, <0.001). In this arm, median percent changes at 96 weeks were significant for hsCRP (−13.9%, *P* < 0.001), IL-6 (−13%, *P* = 0.048), sCD14 (−12.3%, *P* < 0.001), ox-LDL (−31.6%, *P* < 0.001), adiponectin (−13.2%, <0.001) and NT-proBNP (−29.9%, <0.001). There were no significant differences between median percent changes at 48 weeks and median percent changes at 96 weeks in the DTG-I arm.

In the DTG-D arm, median percent changes in biomarkers at 48 weeks were significant for IL-6 (−40.2%, *P* = 0.048) only. In this arm, median percent changes at 96 weeks were significant for hsCRP (−13.5%, *P* < 0.001), IL-6 (−54.6%, *P* < 0.001), sCD14 (−16%, *P* < 0.001), ox-LDL (−25.6%, *P* < 0.001), adiponectin (−13.1%, <0.001) and NT-proBNP (−24.2%, *P* = 0.001).

**Figure 2:**
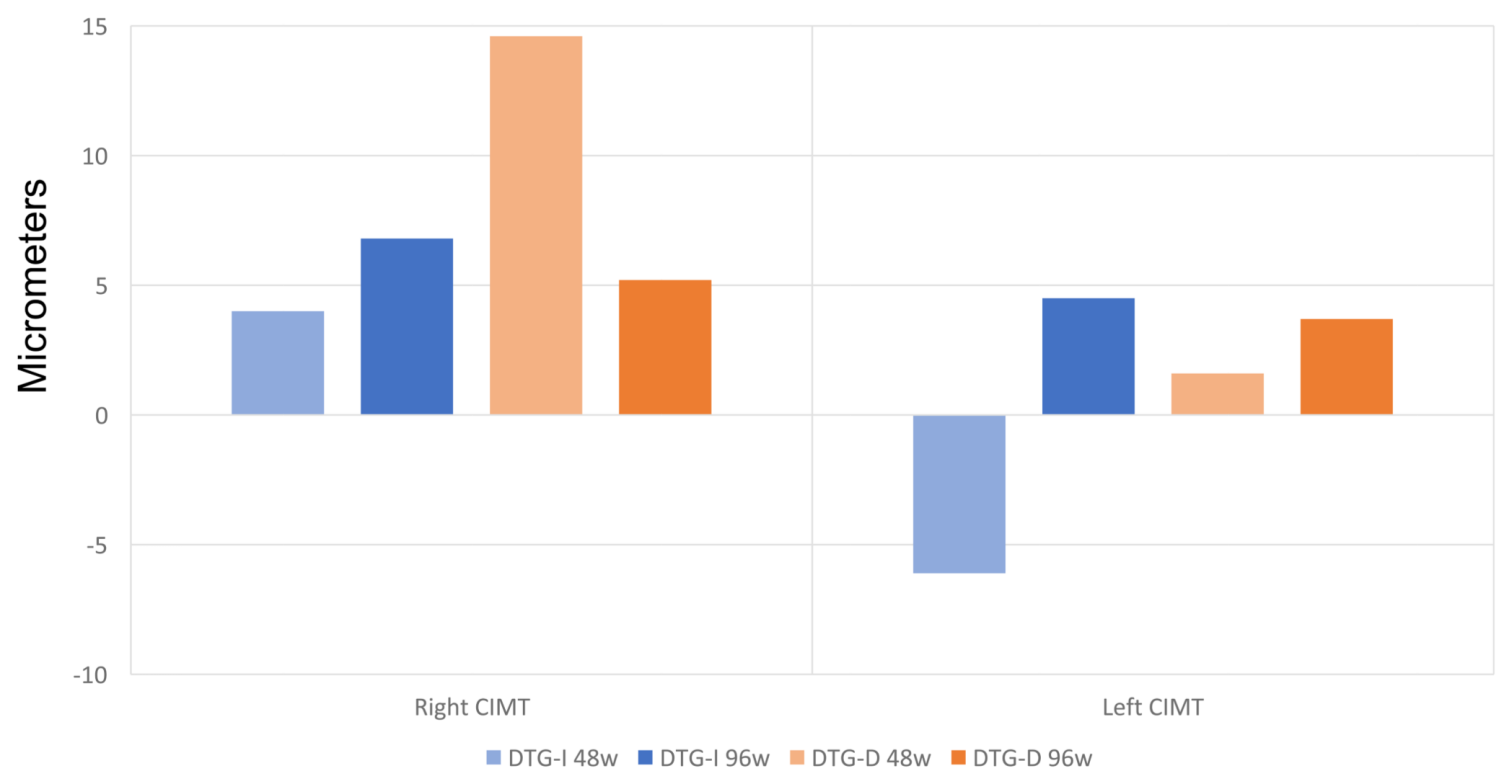
Median changes in right and left CIMT in DTG-I and DTG-D arms at 48 and 96 weeks. There were no significant differences in CIMT progression within arms or between arms at both 48 and 96 weeks. This figure appears in colour in the online version of *JAC* and in black and white in the print version of *JAC*.

In general, CIMT progressed over time (Figure 2). There were consistent favourable, although non-significant, trends on CIMT progression in both carotid arteries in the first 48 weeks of exposure to dolutegravir. Differences within arms or between arms at both 48 and 96 weeks were not significant.

## Discussion

Switching from ritonavir-boosted PIs to dolutegravir in PWH with high cardiovascular risk in the NEAT022 study led to significant bPI-to-DTG switch and subclinical cardiovascular disease reductions at 96 weeks in several plasma biomarkers associated with inflammation, monocyte immune activation, oxidation, insulin resistance and heart failure, but there were no significant changes in CIMT progression at 96 weeks. After the first 48 weeks of exposure to dolutegravir, changes for plasma biomarkers and CIMT progression in the deferred switch (DTG-D) group were roughly consistent with those observed in the early switch (DTG-I) group.

Significant changes observed in sCD14 and adiponectin at 48 weeks in the DTG-I arm were maintained at 96 weeks. Some other biomarkers such as hsCRP, IL-6, ox-LDL, and NT-proBNP, which had decreased but not significantly at 48 weeks, showed significant reductions at 96 weeks. Similar trends were seen when raltegravir was used to replace boosted PIs in the SPIRAL study, in which participants did not need to have a high cardiovascular risk.^20^ Reductions in sCD14 have been consistently reported in other studies switching to dolutegravir plus lamivudine, mostly from boosted PIs but also from other drugs.^21–23^ In NEAT022 there was, however, a persistent reduction in adiponectin (adiponectin was not measured in the SPIRAL study), which is associated with insulin resistance, although we did not measure specific markers of insulin resistance such as homeostasis model assessment-insulin resistance (HOMA-IR) or others. The reduction in adiponectin could be related to higher fat mass as a consequence of the weight gain observed.^12^ However, it is remarkable that insulin itself did not show a significant increase in PWH switching to dolutegravir after 48 or 96 weeks of follow-up. The reduction in plasma lipids and in other plasma biomarkers may offset the clinical consequences of adiponectin reduction on insulin resistance.^24^

There were differences in the progression between right and left common CIMT progression. This observation has been previously reported.^25^ The reason for this phenomenon is not yet clear; however, it is speculated that it may be due to the different origins of the left and right common carotid artery, whereby they are subjected to different flow intensities from the aortic arch. The left common carotid artery stems directly from the arch of the aorta and is affected by aortic arch pressure (hydrostatic pressure). The right common carotid artery stems from the innominate artery, which is an extension of the ascending aorta, and is subjected to significant pressure from the ascending aortic blood flow (dynamic pressure). In addition, the wall shear stress resulting from the interaction between the blood and the intima has previously been associated with development of atherosclerotic plaque, a thick CIMT and vascular structural remodelling.^25^

We were unable to detect changes in the progression of CIMT with the switch from PIs to dolutegravir. Changes were not significant, due at least in part to the high dispersion of CIMT measurements and to the relatively low number of participants in the substudy. A larger sample size and longer follow-up would have been needed to allow for detection of any significant effects associated with this strategy on subclinical cardiovascular benefits.

In summary, switching the boosted-PI component to dolutegravir in virologically suppressed patients with high cardiovascular risk showed significant changes in several plasma biomarkers at 96 weeks. Most of the changes with the dolutegravir switch were favourable, although adiponectin reduction was maintained over time. We were unable to detect significant changes within arms or between arms in the progression of CIMT at 96 weeks.

## Supplementary Material

Supplementary Material
