# Supplementary material for: Long-term effects on subclinical cardiovascular disease of switching from boosted protease inhibitors to dolutegravir
Source: J Antimicrob Chemother. Author manuscript; Available in PMC 2025 Feb 24. (PMC7617418; doi:10.1093/jac/dkad247)
Supplement: Supplementary Material [file EMS202958-supplement-Supplementary_Material.docx]

**Supplementary figure 1.** Study diagram.

**Supplementary figure 2.** Study flow chart.
